# Ergogenic effects of a 10-day L-citrulline supplementation on time to exhaustion and cardiorespiratory and metabolic responses in healthy individuals: a double-blind, randomised, placebo-controlled crossover trial

**DOI:** 10.3389/fspor.2025.1627743

**Published:** 2025-09-05

**Authors:** J. Schierbauer, L. Francis, F. Greco, P. Zimmermann, O. Moser

**Affiliations:** ^1^ Division of Exercise Physiology and Metabolism, Bayreuth Centre of Sport Science, University of Bayreuth, Bayreuth, Germany; ^2^ Department of Movement, Human and Health Sciences, University of Rome Foro Italico, Rome, Italy; ^3^ Interdisciplinary Metabolic Medicine Trials Unit, Department of Internal Medicine, Division of Endocrinology and Diabetology, Medical University of Graz, Graz, Austria

**Keywords:** endurance performance, lactate turnpoint, nitric oxide, cardiac output, cycling

## Abstract

**Introduction:**

L-Citrulline supplementation has been a topic of debate due to its potential to augment L-arginine bioavailability and nitric oxide production. However, it remains uncertain whether it can truly serve as an ergogenic aid in endurance exercise performance. While it was previously recommended to include higher continuous doses of L-citrulline over ≥7 days, this study aimed to investigate whether a relative dosing strategy using 100 mg·kg^−1^ per day over 10 days could improve time to exhaustion (TTE) in healthy young adults.

**Methods:**

Twenty healthy, moderately active adult participants (nine females; age, 24.4 ± 0.9 years; BMI, 24.0 ± 2.5 kg·m^−2^; V̇O_2max_, 43.5 ± 6.3 mL·min^−1^·kg^−1^) received either 100 mg·kg^−1^ per day of L-citrulline (CIT) or a placebo (PLA) for 10 days in a double-blind, randomised, placebo-controlled crossover trial, before they performed two TTE tests at 5% above the second lactate turnpoint (LTP2). Cardiac output (Q̇), oxygen uptake (V̇O_2_), blood glucose ([Glu^−^]) and lactate concentrations ([La^−^]), and rating of perceived exertion (RPE) were quantified during each test.

**Results:**

There was no statistically significant difference in TTE between the trial arms (CIT vs. PLA: 20.5 ± 7.3 vs. 19.8 ± 5.7 min, *p* = 0.43). However, a trend was observed in the female subgroup (24.4 ± 6.2 vs. 21.9 ± 4.8 min, *p* = 0.06). Cardiac output (Q̇) also did not show significant differences between mean (CIT, 18.3 ± 3.7 L·min^−1^; PLA, 19.0 ± 4.5 L·min^−1^; *p* = 0.40) and maximum values (CIT, 28.7 ± 5.7 L·min^−1^; PLA, 26.6 ± 4.9 L·min^−1^; *p* = 0.22) in both trial arms. Mean V̇O_2_ was 1.77 ± 0.28 and 1.84 ± 0.25 L·min^−1^ (*p* = 0.06) for CIT and PLA, respectively. Similarly, mean maximum systolic (165 ± 17 vs. 171 ± 18 mmHg, *p* = 0.23) and diastolic (81 ± 6 vs. 83 ± 6 mmHg, *p* = 0.19) blood pressure levels were not significantly different between CIT and PLA trial arms.

**Discussion:**

Higher continuous doses of L-citrulline over 10 days do not increase TTE in healthy, moderately active individuals when compared with a placebo. Therefore, it is reasonable to assume that L-citrulline does not offer an ergogenic benefit for endurance performance. However, future research may focus on female populations or exercise protocols that involve longer distances to simulate competition.

## Introduction

Nitric oxide (NO) is a signalling molecule associated with improved skeletal muscle function and exercise performance by increasing blood flow to working muscles, enhancing contractility, and improving mitochondrial respiration. NO production can be enhanced by exogenous substances with typical dietary strategies including the consumption of green leafy vegetables and beetroot juice or direct supplementation with L-arginine or L-citrulline (1). L-arginine is part of the human diet, and only 5%–15% of plasma arginine originates from *de novo* synthesis (2, 3). After oral administration, L-arginine is subject to both extensive presystemic and systemic elimination in the gut and liver (4). In contrast, the non-essential amino acid L-citrulline is subject only to systemic metabolism. In this process, L-citrulline is enzymatically converted to L-argininosuccinate and subsequently to L-arginine (5), making it an L-arginine precursor.

In recent years, L-citrulline has gained interest in the field of sports and exercise physiology due to its potential to increase NO activity. NO serves as a vasodilator, widening blood vessels and thereby enhancing blood flow and nutrient delivery to muscles (6). It has therefore been postulated that L-citrulline supplementation may improve exercise performance by delaying fatigue, enhancing muscle oxygenation, and reducing the rating of perceived exertion (RPE) during physical activities (7, 8). Regarding the postulated positive effects on exercise performance, however, research investigating the acute and chronic L-citrulline supplementation in recreational and elite athletes is contradictory. For instance, following multiday L-citrulline supplementation, both significant increases (7 days with 6 g·day^−1^) (9) or no effect (16 days of enriched watermelon juice, ∼3.4 g) (10) regarding time-to-exhaustion protocols were reported. A single bolus of L-citrulline has not been shown to improve time-to-exhaustion or time trial exercise performance, with only limited evidence suggesting an ergogenic effect on high-intensity, high-strength, or high-power exercise performance (8). Accordingly, further studies are needed, especially because L-citrulline is assumed to have positive effects across a wide range of populations, e.g., recreational athletes, resistance-trained populations, aerobic and anaerobic athletes, and even clinical populations (9, 11, 12).

In their latest review on the impact of citrulline supplementation on exercise performance, Łukaszewicz et al. (13) concluded that, although there are a large number of indications for a potential ergogenic effect, it is currently not possible to state unequivocally whether an increased NO production due to citrulline supplementation positively improves the function and efficiency of the muscular system or the efficiency of blood supply to organs and muscles.

It has also been concluded that methodological variations between studies, including differences in dosage, acute vs. chronic supplementation, exercise modalities [time to exhaustion (TTE), time to completion, single set/multi-set resistance training, sprint or power training], form of L-citrulline (such as L-citrulline malate), and training status or sex of the study population, all possibly contribute to inconsistent findings (1, 14, 15).

To date, it remains uncertain which L-citrulline supplementation protocol offers an optimal stimulus for NO production, thus enhancing exercise performance outcomes. Moreover, it has previously been recommended that future studies should include higher continuous doses scaled to body mass and supplemented over a multiday period (>7 days) to further evaluate the topic (16).

To address the aforementioned gaps in the existing literature, this double-blind, randomised, placebo-controlled crossover trial aimed to investigate the ergogenic effect of a 10-day L-citrulline supplementation with a weight-based dose (100 mg·kg^−1^) on time to exhaustion and cardiorespiratory and metabolic responses during a high-intensity constant load cycling exercise in healthy adult individuals.

## Materials and methods

### Ethics approval

The study protocol was approved by the local ethics committee of the University of Bayreuth (Germany) (24-010, 23 April 2024), and the study was conducted in conformity with the Declaration of Helsinki and guidelines for good clinical practice (17, 18). Before any trial-related activities, all potential participants were informed about the study protocol and provided written informed consent.

### Eligibility criteria and assessment of eligibility

The eligibility criteria included male or female individuals aged between 18 and 35 years with a body mass index (BMI) of 18.0–29.9 kg·m^−2^. Participants were excluded if they were simultaneously enrolled in a different study, received any kind of medicinal product, had a blood pressure level outside the range of 90–150 mmHg for systolic and 50–95 mmHg for diastolic after resting for 5 min in a supine position, or had a significant abnormal electrocardiogram screening as determined by a medical investigator. Participants were also excluded if they had metabolic disease, including renal or thyroid, or a history of multiple and/or severe anaphylactic reactions to any trial-related products. Moreover, females of childbearing potential who intended to become pregnant or were pregnant, with the latter being confirmed by a positive pregnancy test, were excluded. The inclusion and exclusion criteria were assessed by the same investigator at the screening visit prior to the start of the study.

### Study design

This was a single-centre, double-blind, randomised, placebo-controlled crossover trial investigating the effects of a 10-day L-citrulline supplementation on time to exhaustion and cardiorespiratory and metabolic responses during a high-intensity constant load cycling exercise in healthy adult participants. This study consisted of one screening visit and two trial visits that were preceded by two 10-day supplementation phases with a minimum of a 7-day washout period between trial visits. The screening visit and two trial visits were performed under standardised laboratory conditions, i.e., a room temperature between 21°C and 22°C and a humidity of 50%.

### Screening visit

During the screening visit, participants were informed about all study-related procedures, received study instructions, and provided informed consent. After inclusion in the study, they were assigned to ascending numbers and then allocated to the order in which the trial visits were conducted following a crossover, randomised fashion using the Research Randomizer® software (1:1) (14). Afterwards, participants were examined for their general health status via a physical examination including an automatic blood pressure measurement (BU 510 Blood Pressure Monitor, Medisana GmbH, Neuss, Germany) and a 12-lead electrocardiogram (ECG) recording after resting for 5 min in a supine position (AMEDTEC ECGpro®, Cardiopart 12, Straessle & Co. Medizintechnik GmbH, Albstadt, Germany). Subsequently, body composition was analysed in duplicate using a bioelectrical impedance analysis (InBody 720, InBody, Seoul, South Korea), and body height was measured manually (Seca 217, Seca, Hamburg, Germany). Participants were then asked about the intensity type of physical activity and sitting time as part of their daily lives using the German version of the International Physical Activity Questionnaire (IPAQ-SF) (15).

To determine the individual exercise intensity for the constant load cycling sessions at the following trial visits, a cardiopulmonary exercise test (CPX) was conducted on a mechanically remote-controlled cycle ergometer (Excalibur Sport, Lode, Groningen, the Netherlands), which involved a 3-min sitting phase followed by a standardised 3-min warm-up at 20 W and 1 min incremental steps (i.e., 20 W·min^−1^ for males and 15 W·min^−1^ for females) until exhaustion (19). Upon exhaustion, the test was concluded with a 3-min cooldown and another 3 min sitting phase on the ergometer.

Minute ventilation (V̇E), tidal volume (V̇T), respiratory rate, respiratory exchange ratio (RER), oxygen uptake (V̇O_2_), and carbon dioxide output (V̇CO_2_) were assessed via a high-resolution, portable spirometry system with breath-by-breath technology (Metalyzer 3B, CORTEX Biophysik GmbH, Leipzig, Germany). The maximally attained V̇O_2_ value, defined as the highest 30 s interval during exercise, is hereby referred to as V̇O_2max_, as plateau criteria were met during the CPX test, e.g., the obligatory plateau of V̇O_2_ (20) and, if necessary, the use of secondary criteria, i.e., maximum lactate concentrations and respiratory exchange ratios.

In addition, blood glucose ([Glu^−^]) and lactate ([La^−^]) concentrations were measured via capillary blood samples collected from the earlobe at rest, after the warm-up, at the end of every incremental step, and at 3 and 6 min postexercise (Biosen S-Line, EKF Diagnostics, Barleben, Germany). Systolic and diastolic blood pressure levels were also manually measured at rest, after the warm-up, every 3 min during exercise, and at 3 and 6 min postexercise. The cardiac response was continuously measured using a 12-lead ECG.

Prior to leaving the lab, participants were informed about the testing day inclusion and exclusion criteria for the upcoming trial visits (see below, V1–V2). They received the first of their two supplements consisting of an individual amount of 100 mg·kg^−1^ body mass per day of either L-citrulline (CIT, Functional Cosmetics Company AG, Muttenz, Switzerland) or cellulose supplements which served as a placebo (PLA, Mikrokristalline Cellulose 102, Getschmann & Getschmann GbR, Augsburg, Germany). The selected dose was informed by prior studies that employed body mass-adjusted L-citrulline supplementation demonstrating performance-enhancing effects (21, 22). Both supplements were supplied as white powders with the same texture and had to be consumed with 200 mL of water every morning upon waking for 9 days. On the 10th and final day, participants had to consume their sample 60 min prior to the exercise test (see below). To ensure a double-blind, placebo-controlled approach, all samples were prepared in single plastic containers—one for each day—and labelled with the participant’s study ID and the corresponding trial visit by a researcher who was not involved in the study procedure. Participants were also asked to keep a digital food diary (FatSecret, Secret Industries Pty Ltd., Caulfield North, Australia) during each 10-day supplementation phase and to replicate their exact food intake on the 2 days prior to the second trial visit and on the day of the visit itself.

### Trial visits (V1–V2)

Before each trial visit, participants were required to refrain from intense exercise for 48 h and remain physically inactive for 24 h. In addition, alcohol consumption was prohibited during the 24 h before the trial visits. At the start of each trial visit, participants were screened based on the inclusion and exclusion criteria. The IPAQ-SF was documented again to control changes in physical activity levels between trial visits. Participants were asked to export the data from their food diaries to check for macronutrient differences (i.e., carbohydrate intake on the days prior to V2 only). Participants were then asked to report any side effects related to supplementation with either CIT or PLA. Female participants were asked about their current menstrual cycle phase and whether their cycle occurs regularly. Participants then had to take their last supplement of either CIT or PLA directly in the lab before a timer was started. Exactly 60 min thereafter, they performed a high-intensity constant load cycling exercise until exhaustion at 5% above their individual LTP2.

The exercise protocol of the time-to-exhaustion (TTE) test started with a 3 min resting phase on the cycle ergometer followed by a 3 min warm-up at 40 W. During the test, participants were asked to maintain a self-selected cadence between 80 and 100 revolutions per minute, which also had to be used during the second TTE test. Exhaustion was defined as cycling below a cadence of 60 revolutions per minute for >3 s. Participants were asked to maintain the target workload for as long as possible and were motivated by strong verbal encouragement. If desired, they were allowed to listen to music of their choice, provided the same music was used during the following trial visit.

Based on the [La^−^] from the CPX test at the screening visit, the first (LTP1) and second lactate turnpoints (LTP2) were assessed using a computer-aided linear regression breakpoint method (Vienna CPX-Tool, University of Vienna, Vienna, Austria) integrating the turn point concept. For a more detailed explanation, see Tschakert and Hofmann (23) and Binder et al. (24).

As with the screening visit, a breath-by-breath analysis was performed to assess the ventilatory parameters during the constant load cycling exercise. In contrast to the CPX test, maximally attained V̇O_2_ values during constant load cycling exercise (again defined as the highest 30 s interval during exercise) are referred to as V̇O_2peak_. In addition, stroke volume (SV), heart rate (HR), and cardiac output (Q̇) were measured continuously during exercise using a portable, battery-powered, and non-invasive cardiac monitoring device with signal morphology-based impedance cardiography (PhysioFlow Enduro, Manatec Biomedical, Paris, France). For a more detailed description of the method, see Charloux et al. (25). Lastly, systolic and diastolic blood pressure levels, [Glu^−^] and [La^−^] concentrations, and rating of perceived exertion (RPE) using the 15-grade Borg scale (26) were measured at rest, after the warm-up, every 3 min during exercise, at exhaustion, and at 3 and 6 min postexercise in the same manner as with the screening visit.

**Figure 1:**
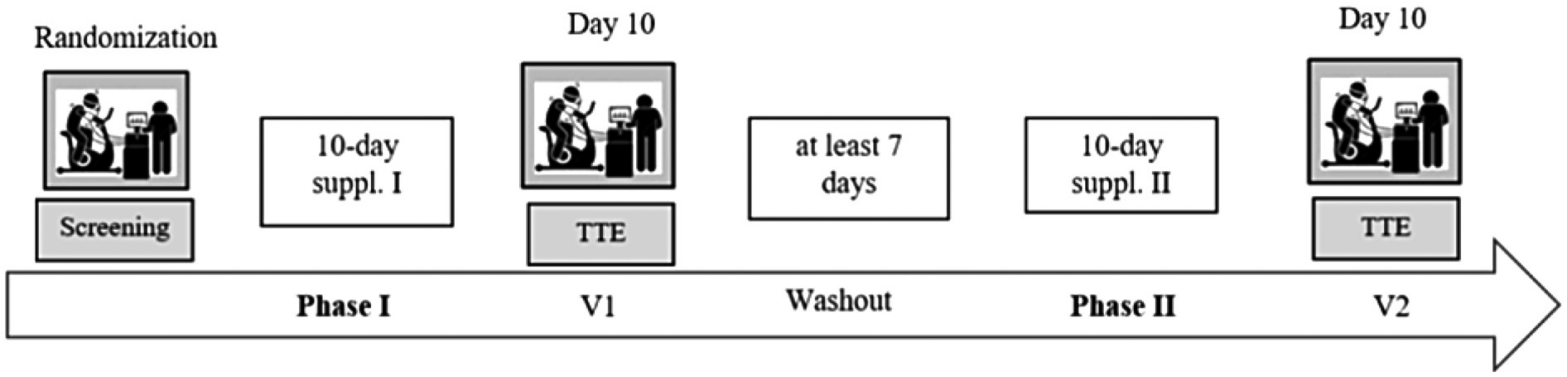
Study flowchart. After a screening visit and the first of two supplementation phases, a TTE test was performed. Then a washout phase had to be maintained (≥7 days) before the second supplementation phase began that concluded with the second TTE test.

To ensure a standardised testing protocol and avoid circadian variation, participants performed each test at the same time of day (at 8 a.m., 10 a.m., or 12 p.m.). Any form of food or fluid intake was permitted during the trial visits. To reduce thermoregulatory variability, participants were asked to wear the same clothing during every trial visit, while a minimum washout period of 7 days had to be maintained between visits. A graphical display of the study design is shown in Figure 1.

### Sample size estimation

An *á priori* sample size estimation was performed using G*Power (3.1.9.2, University of Kiel, Kiel, Germany) based on a similar study project including trained cyclists (27), resulting in an effect size of *d* = 0.69 (TTE in group 1, 47.9 ± 8.2 min; TTE in group 2, 45.4 ± 7.8 min). The level of significance was set at 0.05 and the power (1-*β* error) at 0.80 with a correlation between trial arms of 0.9. This resulted in *a priori* calculated sample size of *n* = 19 per trial arm. Since this is a crossover study, the total number of participants required is also 19.

### Statistical analyses

Data were collected and summarised in a Masterfile in Excel (Microsoft Excel, Microsoft Corporation, Redmond, MA, USA) and analysed in GraphPad Prism (Version 8.0.2, GraphPad Software Inc., Boston, MA, USA). Testing for normal distribution was computed via the Shapiro–Wilk test (passed normality testing at *α* ≥ 0.05), and data are presented according to their distribution as mean or median ± standard deviation, 95% confidence interval, and coefficient of variation (CV). For the primary outcome, TTE for CIT and PLA were compared via a paired *t*-test to find statistically significant differences between trial arms. For the secondary outcomes, and based on the results of the normality testing, either paired *t*-tests or Wilcoxon matched-pairs signed rank tests (i.e., SV, HR, Q̇, and ventilatory parameters) were calculated. A two-way repeated-measures analysis of variance with Tukey's multiple-comparisons test was calculated to find statistically significant differences in [Glu^−^] and [La^−^] concentrations between trial arms (CIT and PLA) and time points (at rest and at exhaustion). Statistical significance was accepted at *p* < 0.05 (two-tailed).

## Results

Twenty healthy young adults were recruited for this study, and none withdrew prematurely. Thus, a total of 20 participants (nine females) were included for statistical analysis. Physical activity measurements assessed before each trial visit were 4,365 ± 2,330 (CIT) and 3,843 ± 2,731 (PLA) total MET-min·week^−1^, with no significant difference between trial arms (*p* = 0.14). Regarding the menstrual cycle of the female participants, all but one performed their trial visits in distinct phases of their respective cycle, with two females being in their follicular phase during one of the two trial visits. All female participants reported being eumenorrheic. Participant characteristics including anthropometrics and body composition are presented in Table 1.

**Table 1:** Participant characteristics (*n* = 20) presented as mean with standard deviation (SD) and 95% confidence interval (CI) including anthropometry and body composition.

Characteristic	Mean ± SD	95% CI of the mean
Age (years)	24.4 ± 0.9	23.9–24.8
Body mass (kg)	73.4 ± 11.3	68.1–78.7
Height (cm)	175 ± 7	171–178
Body mass index (kg·m^−2^)	24.0 ± 2.6	22.8–25.2
Fat mass (kg)	15.8 ± 5.5	13.2–18.3
Fat mass (%)	20.8 ± 7.0	17.5–24.0

### Cardiopulmonary exercise test

Maximum power output (*P*_max_), defined as the last completed increment, was 3.9 ± 0.4 W·kg^−1^ (283 ± 55 W) and ranged between 3.1 and 5.0 W·kg^−1^ (200 and 415 W). Maximum oxygen uptake (V̇O_2max_), defined as the highest 30 s interval during exercise, was 43.5 ± 6.3 mL·min^−1^·kg^−1^. Maximum [La^−^] and respiratory exchange ratio (RER) were 14.2 ± 2.1 mmol·L^−1^ and 1.4 ± 0.1, respectively. Maximum HR was 190 ± 7 beats·min^−1^, respectively.

The calculated mean LTP2 was 203 ± 47 W which equalled 71 ± 4% of *P*_max_. The calculated mean power output for the TTE cycling exercise visits was 213 ± 49 W (75 ± 4% of *P*_max._).

### L-Citrulline and placebo supplementation

Following randomisation, nine participants received CIT first and eleven received PLA. Based on individual body mass, participants received a mean of 7.4 ± 1.1 g·day^−1^ of either CIT or PLA, ranging from 5.4 to 9.1 g·day^−1^. Three participants reported side effects related to the supplementation. One participant reported flatulence in both trial arms (*n* = 1). Other side effects included stomach cramps in the CIT trial arm (*n* = 1) and diarrhoea in the PLA trial arm (*n* = 1), but not within the same participant. Supplementation with both CIT and PLA, however, was completed in full (10/10) by all but one participant (9/10).

### Time to exhaustion

**Figure 2:**
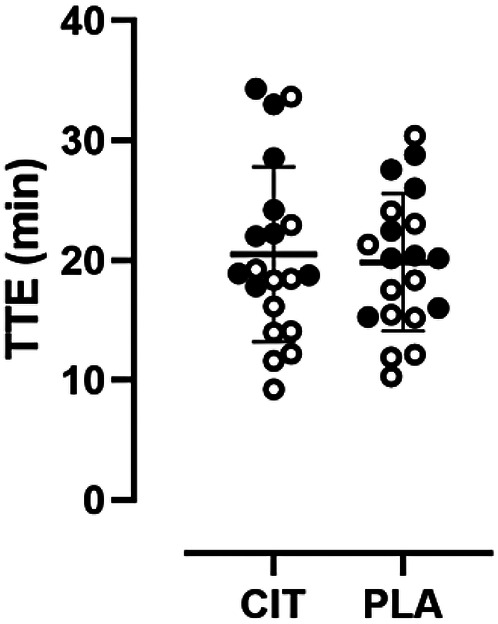
Scatter plot with individual sex-specific TTE values (open circles = males, filled circles = females) in comparison of trial arms.

TTE for both trial arms passed normality testing. There was no statistically significant difference in TTE between the CIT and PLA trial arms (20.5 ± 7.3 vs. 19.8 ± 5.7 min, *p* = 0.43, see Figure 2). An order effect was detected when comparing the TTE between the first and second trial visits (19.2 ± 5.8 vs. 21.1 ± 7.1 min, *p* = 0.01). A sex-specific analysis also did not reveal a statistically significant difference between CIT and PLA trial arms; however, a trend was observed for the female subgroup (males, 17.3 ± 6.7 vs. 18.2 ± 6.1 min, *p* = 0.35; females, 24.4 ± 6.2 vs. 21.9 ± 4.8 min, *p* = 0.06).

### Haemodynamics

**Figure 3:**
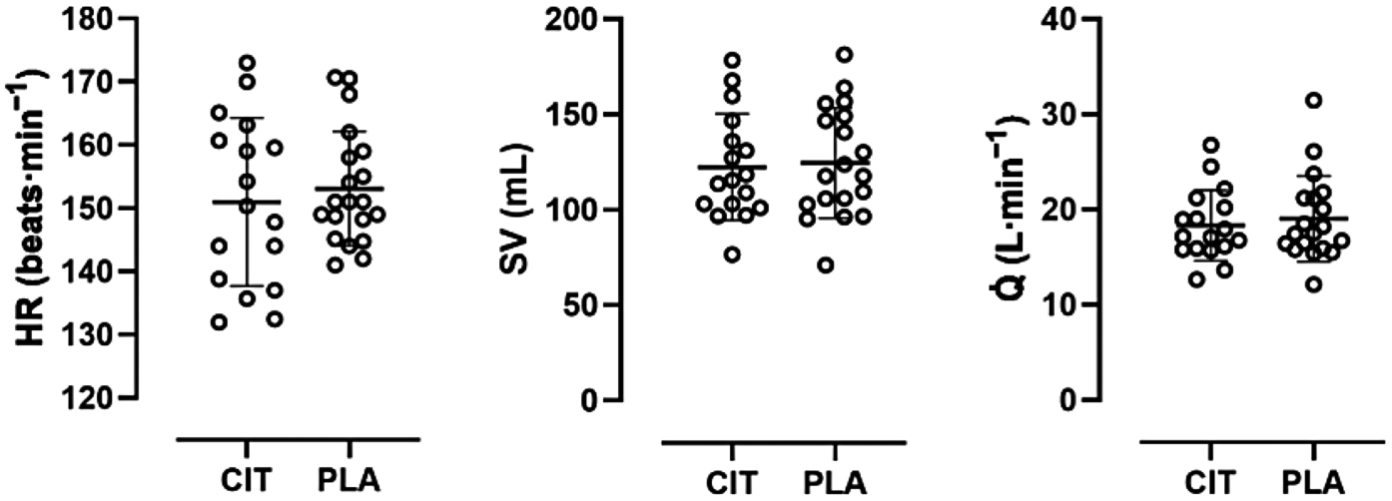
Scatter plot with mean and individual (open circles) HR, SV, and Q̇ values in comparison of trial arms.

All data except for maximum HR were normally distributed. There were no statistically significant differences in mean HR (CIT, 151 ± 13 beats·min^−1^; PLA, 153 ± 9 beats·min^−1^; *p* = 0.22) and maximum HR (CIT, 188 ± 8 beats·min^−1^; PLA, 190 ± 13 beats·min^−1^; *p* = 0.39) between trial arms. For stroke volume, no statistically significant differences were found between mean (CIT, 122 ± 28 mL; PLA, 125 ± 28 mL; *p* = 0.80) and maximum values (CIT, 157 ± 36 mL; PLA, 148 ± 31 mL; *p* = 0.14) in both trial arms. Accordingly, Q̇ did not show significant differences in mean (CIT, 18.3 ± 3.7 L·min^−1^; PLA, 19.0 ± 4.5 L·min^−1^; *p* = 0.40) and maximum values (CIT, 28.7 ± 5.7 L·min^−1^; PLA, 26.6 ± 4.9 L·min^−1^; *p* = 0.22) in both trial arms (see Figure 3).

Mean resting systolic (127 ± 9 vs. 125 ± 12 mmHg, *p* = 0.42) and diastolic (79 ± 4 vs. 81 ± 5 mmHg, *p* = 0.12) blood pressure levels were also not significantly different between CIT and PLA trial arms. The same was found for maximum values at exhaustion (systolic, 165 ± 17 vs. 171 ± 18 mmHg, *p* = 0.23; diastolic, 81 ± 6 vs. 83 ± 6 mmHg, *p* = 0.19).

### Glucose and lactate

All data passed normality testing. Mean [Glu^−^] at rest was 88.0 ± 5.9 and 89.5 ± 6.6 mg·dl^−1^ for CIT and PLA, respectively. Mean [Glu^−^] at exhaustion was 94.2 ± 18.9 and 92.9 ± 16.3 mg·dl^−1^ for CIT and PLA, respectively. A two-way repeated-measures analysis of variance showed that there were no statistically significant differences between the trial arms under both resting (*p* = 0.82) and postexercise conditions (*p* = 0.76). The mean postexercise [Glu^−^] values also were not significantly different compared with the respective resting values (CIT, *p* = 0.33; PLA, *p* = 0.81).

Mean [La^−^] at rest was 1.01 ± 0.22 and 1.01 ± 0.29 mmol·L^−1^ for CIT and PLA, respectively. Mean [La^−^] at exhaustion was 11.96 ± 2.66 and 12.31 ± 2.97 mmol·L^−1^ for CIT and PLA, respectively. There were no statistically significant differences between the trial arms under both resting (*p* = 0.99) and postexercise conditions (*p* = 0.18).

### Ventilatory measurements and rating of perceived exertion

Peak V̇O_2_ during constant load cycling was 37.4 ± 3.1 mL·min^−1^·kg^−1^ (CIT) and 36.0 ± 4.1 mL·min^−1^·kg^−1^ (PLA, *p* = 0.26). Mean V̇CO_2_ was 2.19 ± 0.34 L·min^−1^ (CIT) and 2.19 ± 0.36 L·min^−1^ (PLA, *p* = 0.43). Mean RPE was 16.2 ± 0.8 (CIT) and 16.3 ± 0.7 (PLA, *p* = 0.77). For further information, see Table 2.

**Table 2:** Ventilatory and RPE values presented as mean ± standard deviation, 95% confidence interval, and coefficient of variation (CV) during constant load cycling exercise (TTE) in comparison of trial arms.

Parameter	Trial arm	Mean ± SD	95% CI	CV (%)	*P*-value
V̇O_2peak_ (mL·min^−1^·kg^−1^)	CIT	37.4 ± 3.1	33.6–41.3	8.3	0.26
	PLA	36.0 ± 4.1	30.9–41.2	11.5	
V̇O_2_ (L·min^−1^)	CIT	1.77 ± 0.28	1.42–2.12	15.9	0.06
	PLA	1.84 ± 0.25	1.52–2.16	14.0	
V̇CO_2_ (L·min^−1^)	CIT	2.19 ± 0.34	2.02–2.35	15.9	0.82
	PLA	2.19 ± 0.36	2.02–2.36	16.8	
RER	CIT	1.11 ± 0.04	1.09–1.14	4.3	0.87
	PLA	1.11 ± 0.12	1.06–1.17	10.8	
V̇E (L·min^−1^)	CIT	72.4 ± 17.1	64.4–80.4	23.6	0.30
	PLA	75.1 ± 11.5	69.7–80.5	15.3	
V̇T (L·min^−1^)	CIT	1.98 ± 0.37	1.80–2.16	18.9	0.41
	PLA	2.01 ± 0.41	1.81–2.21	20.8	
Respiratory rate (breaths·min^−1^)	CIT	36.1 ± 5.1	33.7–38.5	14.2	0.67
	PLA	35.9 ± 6.2	32.9–38.8	17.5	
O_2_ pulse (mL·beat^−1^)	CIT	12.6 ± 2.9	11.1–14.0	23.4	0.31
	PLA	12.0 ± 2.2	11.0–13.0	18.1	
V̇E/V̇O_2_ (L·min^−1^)	CIT	35.9 ± 3.7	34.2–37.7	10.2	0.37
	PLA	36.1 ± 3.1	34.6–37.6	8.6	
V̇E/V̇CO_2_ (L·min^−1^)	CIT	32.1 ± 2.8	30.8–33.4	8.6	0.44
	PLA	32.7 ± 2.7	31.4–34.0	8.4	
RPE (Borg)	CIT	16.2 ± 0.8	15.8–16.6	4.7	0.51
	PLA	16.3 ± 0.7	16.0–16.7	4.6	

## Discussion

This double-blind, randomised, placebo-controlled crossover trial aimed to investigate the ergogenic effect of a 10-day L-citrulline supplementation on TTE during a high-intensity constant load cycling exercise in healthy, moderately active individuals. Our results demonstrated supplementation with 100 mg·kg^−1^ L-citrulline over a period of 10 days did not improved TTE. Furthermore, cardiac output and ventilatory measurements did not show significant differences when compared with the placebo trial arm.

To date, only a few studies have investigated the multiday effect of L-citrulline supplementation on TTE or time trial performance (16). With regard to the testing protocol, we chose TTE since, in this context, it has been postulated that multiday supplementation of >7 days may improve the likelihood of enhancing endurance performance-related outcomes (8); however, the results offer equivocal findings. For instance, Bailey et al. (9) showed a significant increase in TTE during moderate-intensity cycling exercise in recreationally active men after 7 days of supplementation with 6 g·day^−1^ L-citrulline. Suzuki et al. (28) found that after supplementation with 2.4 g·day^−1^ in healthy trained men, time-to-completion and power output during a 4 km cycle time trial were significantly improved. In contrast, Bailey et al. (10) reported no improvements in TTE or blood lactate kinetics in healthy recreationally active adult males despite improved NO bioavailability during moderate- and severe-intensity cycling exercise after 16 days of 3.4 g·day^−1^ L-citrulline supplementation. These findings were also confirmed by a study from Shanely et al. (29), who found no ergogenic effect on time-to-completion and mean power output during a 75 km cycle time trial after 14 days of ∼1.5 g·day^−1^ L-citrulline supplementation. These results, along with our findings, are in line with a recent meta-analysis by Harnden et al. that suggested no significant benefit of L-citrulline supplementation for endurance performance. However, the authors stated that, given the small evidence base, further research should include higher and continuous doses over at least 7 days to fully evaluate this topic. We tried to implement both recommendations within this study by using a higher continuous dose over a total of 10 days. In addition, we would like to point out that this is one of the few studies that also used an individualised approach regarding L-citrulline dosing by administering L-citrulline per kilogram body mass. Considering these methodological characteristics and the results of our study, it can be concluded that even higher doses over longer periods of time also do not offer an ergogenic benefit to endurance exercise performance.

We also found no significant differences in RPE, blood lactate, or haemodynamic measures, i.e., SV, HR, or V̇O_2_. This is intriguing since the potential for L-citrulline to increase NO bioavailability and thus improved fatigue resistance has been postulated multiple times in previous research (8, 16). The underlying mechanisms include improved blood circulation and oxygen delivery to working muscles along with greater oxygen extraction fractions. However, we did not detect an effect of an increased central blood flow, which can be used as an indirect estimate of blood flow to working muscles (30) when analysing SV or oxygen uptake between CIT and PLA. In the study by Bailey et al. (9), it was demonstrated that during severe-intensity exercise, overall V̇O_2_ kinetics were faster following L-citrulline supplementation compared with a placebo. This was explained by an increased NIRS-derived tissue oxygenation index throughout the exercise bout, which suggests that CIT supplementation improved the oxygen delivery to the muscle microvasculature, which, in turn, would permit a greater V̇O_2_ over the initial stages of severe-intensity cycle exercise. It remains unclear why our findings contrast with those of Bailey et al., although the supplementation period and dosage were comparable. In this context, the authors state that these conflicting results might be the consequence of different exercise tests. This is also supported by Gough et al. (1) who reported that the lack of ergogenic effects after citrulline supplementation might stem from exercise protocols featuring a predominantly anaerobic energy contribution. Although this seems reasonable from a strict physiological perspective given the proposed ergogenic mechanisms, we used an exercise intensity with a large aerobic component at a calculated mean power output of 75% of *P*_max_. Moreover, with cycling exercise, we chose a movement that recruits large muscle masses rather than isolated muscle action, which was proposed as a limiting factor in the absence of an ergogenic effect after supplementation (1). Here, the enhanced NO synthesis leading to vasodilation should lead to an improved delivery of blood flow, thus increasing oxygen delivery to and from the working skeletal muscles (31). This mechanism, however, has recently been questioned for single-joint resistance exercise using the near-infrared spectroscopy (NIRS) technique that allows blood flow quantification (32, 33). Although a reliable technique (34), it is still debated whether NIRS is sensitive enough to detect changes in blood flow following citrulline intake.

One of the many factors influencing the ergogenic effect of L-citrulline is training status. In their review, Gentilin et al.(35) pointed out that any improvement in athletic performance is lower in already trained individuals, with the effect becoming smaller the more trained an individual is. The authors argued that it is reasonable to expect that any advantage in NO bioavailability, due to chronic L-citrulline supplementation, may be less evident in the trained population. However, they also mentioned that the chronic intake of 6 g·day^−1^ of L-citrulline improved long-lasting, high-intensity performance regardless of training status and age, indicating a dose-dependent effect. These findings cannot be confirmed by the results of our study. Although our participants can be best described as moderately active, which was mirrored by the IPAQ results, we did not observe a performance-enhancing effect after a higher and longer L-citrulline supplementation. Therefore, it remains controversial whether chronic supplementation of >5 g·day^−1^ of L-citrulline leads to consistent improvements in endurance exercise performance. Previous research has shown that L-citrulline seems to be well-tolerated at doses up to 15 g·day^−1^ in healthy subjects (36), which is why future studies should investigate the effect of higher doses, if any, on NO bioavailability and endurance exercise performance.

In contrast, it was also stated that L-citrulline may only be relevant for competitive athletes, in which outcomes are decided by small margins (33), leaving the discussion controversial. In conclusion, we agree with the arguments provided by Gough et al. (1), who stated that the lack of positive effects within the existing literature is due to several factors, including various testing protocols, the lack of aerobic energy contribution, or the dosing amounts and timing.

### Strengths and limitations

We aimed to recruit healthy young adults for this study and reported specific inclusion and exclusion criteria to reduce the risk of bias selection. However, due to the single-centre nature of the trial, participants were selected from a small population in one location, thus reducing the generalisability of the results to the target population (16). Confounding variables such as fatigue from recent exercise, differences in nutrient content between trial visits, and influence from alcohol or other supplementations were controlled in this study. Participants were asked to document and replicate their dietary intake in the days before the last trial visit and refrain from alcohol and physical exercise before each trial visit. Moreover, due to the crossover design, individual characteristics were unlikely to affect the comparison between citrulline and the control, while the randomisation ensured a reduction of the risk of allocation bias. Our primary outcome measure, i.e., TTE, is measured in a way that reduces the risk of reporting bias. In addition, we reported outcomes for all randomised participants, reducing the risk of attrition bias. The risk of performance bias was also reduced by the double-blind approach. In addition, our methods included impedance cardiography that allows for the determination of central blood flow and thus a more holistic approach to physiological regulatory mechanisms.

However, this study is not without limitations. First, we did not directly measure plasma citrulline and arginine concentrations. Therefore, we cannot tell whether the dosage used in this study was too small to elicit changes in plasma concentrations, thus explaining a potential lack of performance-enhancing effect. However, a study by Bailey et al. (9) has found significantly increased citrulline serum concentrations (and a trend towards an increase in serum NO) after consuming 6 g·day^−1^ for 7 days. This indicates that elevated citrulline (and NO) serum levels should have also been present in this study, given the higher (7.4 g·day^−1^) and longer (10 days) supplementation phase. Second, we found an order effect when comparing the TTE of the first and second trial visits, which is most likely due to a “learning effect” making our participants perform better on the second trial visit. Although we used randomisation as counterbalancing, the lack of a familiarisation visit for the TTE protocol must be regarded as a limitation when interpreting our results. To avoid this in future investigations, participants should be familiarised with all performance tests prior to the experimental testing. Moreover, it has been repeatedly demonstrated that the menstrual cycle in females, e.g., the early follicular phase, has a detrimental effect on various performance outcomes (37). In this study, only two out of nine females were in the follicular phase during one of their trial visits (female 1 during CIT, female 2 during PLA), but with no informative differences in TTE between CIT and PLA (female 1, 18.8 vs. 16.0 min; female 2, 33.0 vs. 28.8 min). If at all, the shorter TTE during PLA in female 2 might be explained by the follicular phase. In contrast, a study by Gonzales et al. (38) indicates that exercise-onset vasodilator kinetics are unaltered after L-citrulline supplementation for 7 days with 6 g per day irrespective of menstrual cycle phase; however, no effect on exercise performance was measured. To date, no female-only samples have been recruited for studies investigating the effect of L-citrulline supplementation on endurance exercise performance. However, it has been suggested that physiological function and response to supplementation may differ according to sex, due to the structural and morphological differences between males and females (39). Since we found a trend towards a significant effect of L-citrulline supplementation on TTE after a sex-specific analysis, future studies should recruit female-only samples and incorporate the phases of the menstrual cycle when scheduling trial visits for female participants, although our results must generally be interpreted with caution given the small sample size of the female subgroup.

## Conclusion

A 10-day L-citrulline supplementation with 100 mg·kg^−1^·day^−1^ does not improve time to exhaustion during a high-intensity constant load cycling TTE test in healthy adult participants when compared with a placebo. From a physiological perspective, haemodynamic measures, e.g., SV, HR, and oxygen uptake, did not differ between trial visits. Taking these and previous findings into account, it can be concluded that L-citrulline supplementation is unlikely to confer any performance benefits for endurance exercise.

## Data Availability

The raw data supporting the conclusions of this article will be made available by the authors, without undue reservation.
